# Isolation, Purification, and Characterization of Fungal Laccase from *Pleurotus* sp.

**DOI:** 10.4061/2011/248735

**Published:** 2011-09-29

**Authors:** Sunil S. More, Renuka P. S., Pruthvi K., Swetha M., S. Malini, Veena S. M.

**Affiliations:** ^1^Department of Biochemistry, Center for Post Graduate Studies, Jain University, 18/3, 9th Main Jayanagar, 3rd Block Bangalore 560011, Karnataka, India; ^2^Department of Biotechnology, Sapthagiri College of Engineering, Bangalore 560090, India

## Abstract

Laccases are blue copper oxidases (E.C. 1.10.3.2 benzenediol: oxygen oxidoreductase) that catalyze the one-electron oxidation of phenolics, aromatic amines, and other electron-rich substrates with the concomitant reduction of O_2_ to H_2_O. They are currently seen as highly interesting industrial enzymes because of their broad substrate specificity. A positive strain was isolated and characterized as nonspore forming Basidiomycetes *Pleurotus* sp. Laccase activity was determined using ABTS as substrate. Laccase was purified by ionexchange and gel filtration chromatography. The purified laccase was a monomer showed a molecular mass of 40 ± 1 kDa as estimated by SDS-PAGE and a 72-fold purification with a 22% yield. The optimal pH and temperature were 4.5 and 65^*°*^C, respectively. The *K*_*m*_ and *V*_max_ values are 250 (mM) and 0.33 (*μ*mol/min), respectively, for ABTS as substrate. Metal ions like CuSO_4_, BaCl_2_, MgCl_2_, FeCl_2_, ZnCl_2_ have no effect on purified laccase whereas HgCl_2_ and MnCl_2_ moderately decrease enzyme activity. SDS and sodium azide inhibited enzyme activity, whereas Urea, PCMB, DTT, and mercaptoethanol have no effect on enzyme activity. The isolated laccase can be used in development of biosensor for detecting the phenolic compounds from the effluents of paper industries.

## 1. Introduction

Laccases (EC 1.10.3.2; benzenediol: oxygen oxidoreductases) are multicopper enzymes belonging to the group of blue oxidases that catalyses oxidation of a wide variety of organic and inorganic compounds, including diphenols, polyphenols, diamines, and aromatic amines. One electron at a time is removed from the substrate, and molecular oxygen is used as the electron acceptor [1]. The substrate loses a single electron and forms a free radical. The unstable radical undergoes further nonenzymatic reactions including hydration, disproportionation, and polymerization [2]. The substrates of laccases may vary from diphenols and polyphenols to diamines, aromatic amines, benzenethiols, and substituted phenols [3]. Laccases are common enzymes in nature, especially in plants and fungi. Besides fungal laccases, which are the most frequently studied form of laccase, bacterial laccases from *Pseudomonas putida F6*, *Pseudomonas *sp. LBC1, and *Escherichia coli* have also been purified and characterized [4]. Most of the laccases studied are of fungal origin especially from the classes of white-rot fungi. Fungal laccases play an important role in plant pathogenesis, pigment production, and degradation of lignocellulosic materials [1, 2]. White-rot basidiomycetes are microorganisms able to degrade lignin efficiently. However, the degree of lignin degradation with respect to other wood components largely depends on the environmental conditions and the fungal species involved. The ability of laccase producing microorganisms or purified laccases to eliminate a wide range of pollutants is currently one of the most interesting subjects for researchers in environmental biotechnology [5]. Laccases can be used in detection of catecholamine neurotransmitters such as dopamine, norepinephrine, [6], laccase-oxidized form of morphine in the presence of codeine by coupling its oxidation with glucose dehydrogenase has been studied [7]. Some fungal laccases degrade toxic fungal metabolites, such as aflatoxin B1 [8], and are also useful in the field of food microbiology. Ethanol is produced from pulp, manufacturing of lightening cream, wine clarification, and is also used in industrial applications such as an oxidizing biocatalyst [6, 9]. Potential application of laccases include textile dye bleaching, pulp bleaching, effluent detoxification, biosensors, and bioremediations [1, 10]. However, a serious problem often encountered with industrial exploitation of fungal laccases is the low production level by the native hosts. This problem may be overcome by heterologous production in fungal hosts capable of producing high amounts of extracellular enzymes generally *Trichoderma reesei *or *Aspergillus *sp. 

The aim of the present paper is to screen and isolate laccase-producing fungi from soil samples using laccase enzyme indicators [11] and identifying a new source of extracellular laccase. The extracellular fungal laccase in the culture medium was subjected to purification, biochemical characterization, and its substrate specificity studies.

## 2. Materials and Methods

Chemicals: all chemicals were of the GR grade, (ABTS), syringaldazine were from Sigma (St. Louis, Mo), 2,6-dimethoxyphenol (DMOP), *o*-toluidine were from Fluka (Switzerland). DEAE-Cellulose and Sephadex G-100 (Pharmacia Biotech, Uppsala, Sweden), Protein Molecular weight markers (Bangalore Genei, Pvt, Ltd).

### 2.1. Isolation of Microorganism

Samples of decomposed stumps, leaf litter, biowaste of municipal compost and fruiting bodies of mushrooms were collected in sterile plastic bags from Lalbagh Botanical Garden, Banerghatta National Park, and saw mill. Samples were inoculated on culture medium (B and K agar) after processing and subcultured to obtain a pure culture. The pure cultures were tested for the presence of laccase using *α*-naphthol. Laccase oxidizes *α*-naphthol to a deep purple complex, giving a visual confirmation for the presence of the enzyme.

### 2.2. Laccase Enzyme Assay

Laccase activity was determined by the oxidation of ABTS method [12]. The nonphenolic dye ABTS is oxidized by laccase to the more stable and preferred state of the cation radical. The concentration of the cation radical responsible for the intense blue-green color can be correlated to enzyme activity and is read at 420 nm [13]. The assay mixture contained 0.5 mM ABTS, 0.1 M sodium acetate (pH 4.5), and a suitable amount of enzyme. Oxidation of ABTS was monitored by determining the increase in A420 (*ε*420, 3.6 × 10^4^ M^−1^·cm^−1^). The reaction mixture contained 0.5 mM substrate (ABTS), 2.8 mL of 0.1 M sodium acetate buffer of pH 4.5, and 100 *μ*L of culture supernatant and incubated for 5 min. Absorbance was read at 420 nm in a spectrophotometer against a suitable blank. One unit was defined as the amount of the laccase that oxidized 1 *μ*mol of ABTS substrate per min. Protein concentration was determined by the dye-binding method of Bradford using BSA as standard [14].

### 2.3. Plate Assay

The laccase plate assay allowed rapid determination of the presence of laccase in the extracellular fluid. 15 mL of sterile agarose (0.5%) medium containing 0.5 mM of ABTS per mL in sodium acetate buffer (pH 4.5, 0.1 M) was placed on a glass plate (5 × 5 cm). The development of an intense bluish-green color around the wells was considered as a positive test for laccase activity [15].

### 2.4. Purification of Enzyme

Purification of Laccase was carried out by the method of Chefetz et al. [16]. The culture filtrate was first filtered and centrifuged at 5000 rpm, supernatant was then subjected to ammonium sulfate precipitation. The precipitate obtained was dialyzed and lyophilized and then loaded onto a DEAE-Cellulose anion-exchange column 1.5 × 18 cm, equilibrated with 10 mM sodium acetate buffer (pH 4.5), with a linearly increasing NaCl concentration gradient (0 to 0.5 M) in the same buffer. The six fractions containing laccase activity were pooled, concentrated, and dialyzed overnight against same buffer. Gel filtration chromatography was performed using sephadex G-100 column 2.0 × 40 cm. The DEAE-purified sample was loaded on to the column and 3 mL fraction were collected. The eluted active fractions were dialyzed and protein content was determined by Bradfords method [8] with crystalline bovine serum albumin as the standard.

### 2.5. Gel Electrophoresis

To determine the purity of the protein and its molecular weight, sodium dodecyl sulfate-polyacrylamide gel electrophoresis [17] was performed with a 12% polyacrylamide gel and protein was visualized by staining the gel with silver staining [18] for determining homogeneity and relative molecular mass using standard molecular weight markers (Genei India Pvt. ltd). 

Native PAGE was performed at room temperature with 12% gel according to the Davis 1964 [19]. After electrophoresis, the slab gels were subjected to the activity staining for the laccase and it was fixed with a solution containing 10% (vol/vol) acetic acid and 40% methanol (vol/vol). The gel was then stained by 300 mM ABTS solution for 5 min for color development. The bands of protein that were associated with laccase activity were seen as green bands in a white background [20].

### 2.6. Laccase Characterization

Optimum temperature and pH were determined by performing enzymatic assays at different temperatures (20–80°C) and pH levels (3–8), respectively. The pH level was adjusted using the following buffers: 0.1 M citrate buffer (pH 3–5), 0.1 M phosphate buffer (pH 6–8), and 0.1 M carbonate buffer (pH 9). The stability of the purified laccase at various temperatures was investigated by preincubating the purified laccase at different temperatures between 4 and 70°C for 1 h, followed by determination of the residual activity. The effect of pH on the laccase stability was determined by incubating the purified enzyme at 4°C in different pH levels for 24 h and determining the residual activity. Substrate concentration ranges of 50–500 l M, 500–1500 l M, and 1000–2500 l M were used for kinetic studies against ABTS, DMP, respectively. The effects of metal ions including Cu^2+^_,_ Ba^2+^_,_ Mg ^2+^_,_ Mn^2+^_,_ Fe^2+^_,_ and Hg^2+^ and inhibitors (EDTA, 1 and 10 mM; NaN_3_, DTT, Urea, and SDS on laccase activity were investigated by incorporating them in to assay mixture prior to determination of residual activity [21].

#### 2.6.1. Kinetic Constants

The reaction rate was determined at five different substrate (2,6-DMP, syringaldazine, and ABTS) concentration in the range of 0.01 to 10 mM. All assays were performed in triplicate. The kinetic constants (*K*_*m*_, and *V*_max_) for purified laccase using DMP as a substrate [21].

## 3. Results

### 3.1. Screening for Laccase Positive Strains

In all, 28 samples were isolated which included *Aspergillus *sp*., Rhizopus *sp.,* Fusarium *sp., *Penicillium *sp.*, Alternaria *sp., and few unidentifiable fungal strains out of which one fungal strain gave positive reaction with *α*-naphthol indicating it to be a laccase producer. The positive strain was found to be a nonspore forming Basidiomycetes with clamp connection, and tentatively identified as *Pleurotus *sp. by Agarkar Research Institute, Pune (India).

### 3.2. Laccase Activity Assay

**Figure 1:**
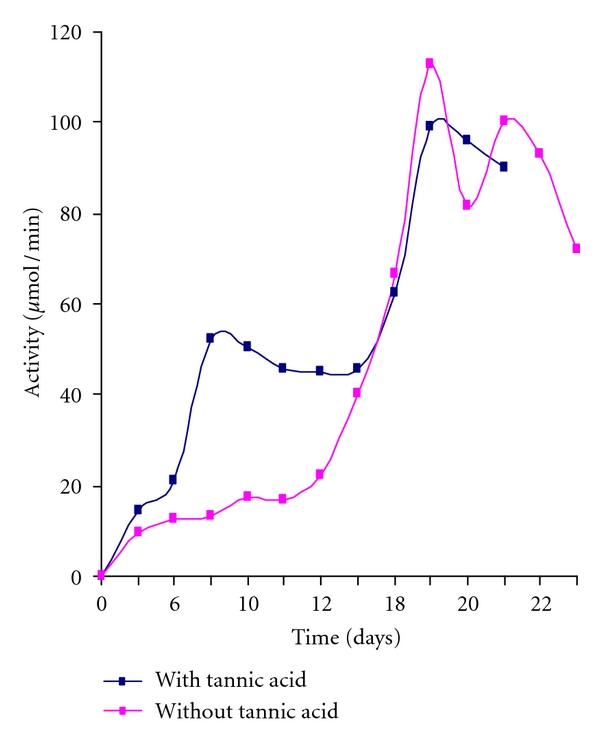
Rate of laccase activity at pH 6.5 with and without inducer.

Laccase activity in the medium reached a maximum on the 19th day. The activity was 83.83 U/mL at pH 5.8 with inducer (Tannic acid) and was 112.88 U/mL at pH 6.5 without inducer (Tannic acid) at room temperature (Figure 1).

#### 3.2.1. Plate Assay

**Figure 2:**
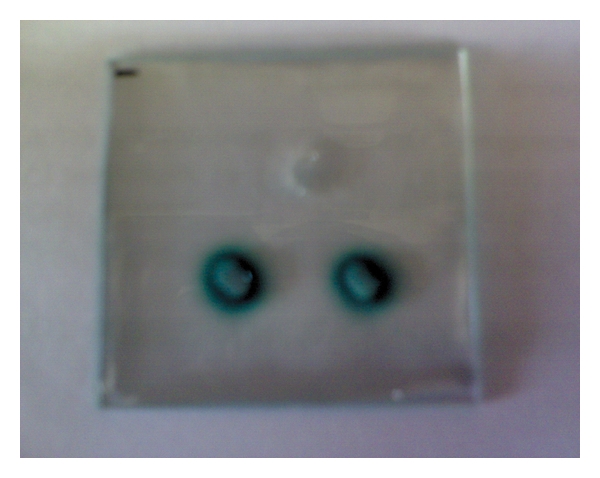
Plate assay for demonstration of laccase activity in purified sample.

The laccase plate assay showed the presence of laccase activity in the partially purified sample. The heat-treated sample showed less intense green color than that of the partially purified sample as shown in (Figure 2).

### 3.3. Purification of Extracellular Laccase 

**Figure 3:**
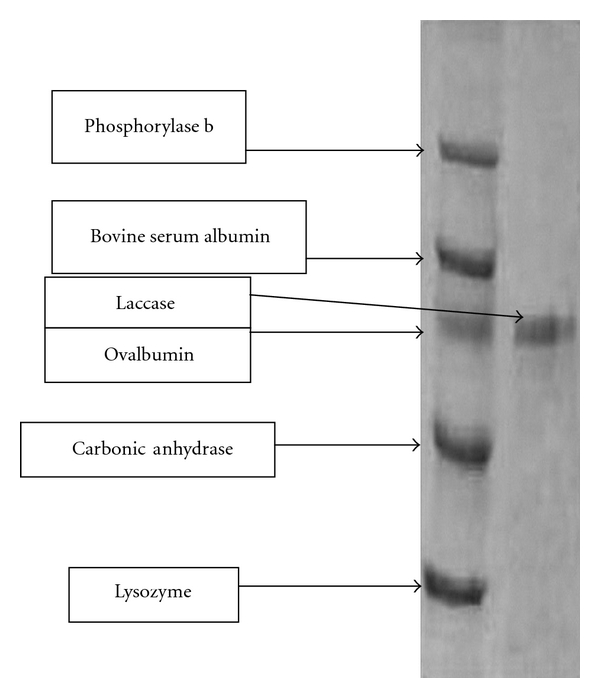
SDS-PAGE of purified laccase from *Pleurotus *sp.

**Figure 4:**
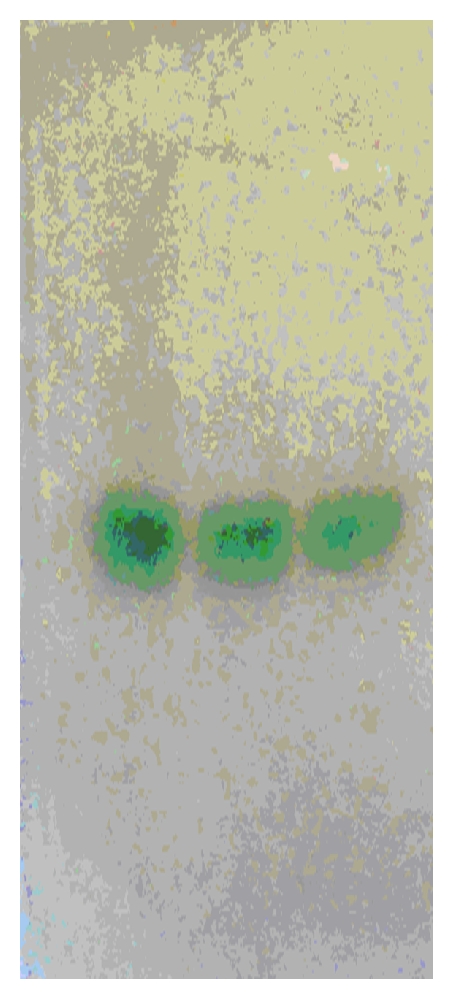
Activity staining of native PAGE gel.

The extracellular laccase from *Pleurotus *sp. was purified to 72.2-fold with a yield of 22.4% (Table 1), using a series of purification steps that included ammonium sulphate precipitation, DEAE-Cellulose column chromatography, and gel permeation using sephadex G-100 column chromatography. The purified enzyme was homogenous showing a single-protein band on SDS-PAGE with a molecular mass of 40 ± 1 kDa when compared to authentic standards (Figure 3). Activity staining of crude and purified enzyme showed that only one extra cellular laccase is secreted (Figure 4). The molecular weight of the purified laccase was further confirmed to be 40 ± 1 kDa by Gel chromatography using sephadex G-100 which was compared with known standard proteins (data not shown).

### 3.4. The Effect of pH and Temperature on Purified Laccase

**Figure 5:**
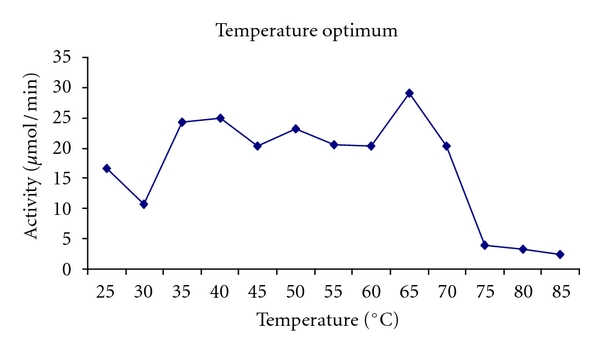
Temperature optima with ABTS as substrate.

**Figure 6:**
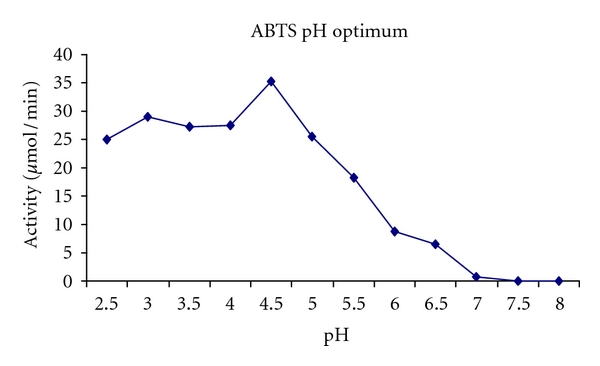
Optimum with ABTS as substrate.

The purified laccase was active in broad pH range of 3–5 with optimum activity at pH 4.5 (Figure 5). The purified laccase has a broad temperature sensitive 35–70°C and the optimum temperature for the laccase was observed at 65°C (Figure 6). Temperature kinetics of the laccase suggests that the enzyme activity increases sharply from 60 to 65°C followed by a decline after 70°C. The laccase was stable in at 60°C for 8 hrs. The enzyme at 75°C was stable up to 30 min, and after 90 min it retained 38% of the activity. The purified enzyme at room temperature was stable for 20 days and stable for 60 days when stored at −4°C.

### 3.5. Kinetic Studies

**Figure 7:**
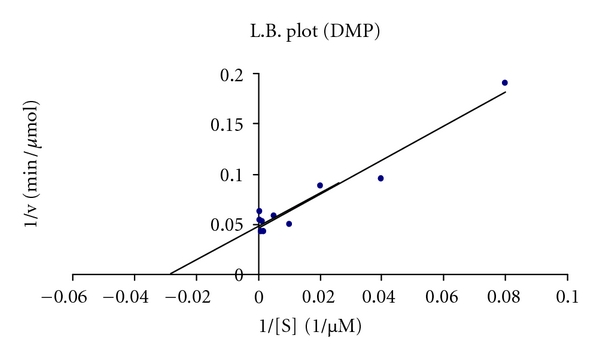
Lineweaver-Burk plot with 2,6-DMP as substrate.

The *K*_*m*_ and *V*_max_ values for purified laccase were 250 (mM) and 0.33 (*μ*mol/min), respectively, for ABTS as substrate where as *K*_*m*_ values for phenolic compound 2,6-DMP were 38.46 mM and *V*_max_ 20 (*μ*mol/min) which shows that phenolic compounds are better substrates for purified laccase (Figure 7).

### 3.6. The Effect of Metal Ions and Inhibitors/Group Specific Reagents

Zinc inactivated the enzyme completely at 2 mM concentration. Nearly 60% activity was lost in the presence of 2 mM of Mn^2+^_,_ Hg^2+^ and Fe^2+^ (Table 2). None of the metal ions stimulated the laccase activity. Sodium azide was a potent inhibitor of enzyme, inactivated the purified laccase completely, and nearly instantaneously with a ki value of 0.03 mM compared to that of EDTA which had moderately inhibited the purified laccase with an ki value of 3.2 mM. At 0.1% SDS concentration, complete activity was lost, hence it was much more effective denaturant. The Urea (5 M), mercaptoethanol, DTT, and PCMB did not inhibit the enzyme (Table 3).

## 4. Discussion

Laccases were first described in 1883 from the Japanese lacquer tree *Rhus vernicifera *[22]. Since then, several laccases have been studied with respect to their biological function, substrate specificity, copper binding structure, and industrial applications [1, 2, 23]. *Pleurotus *sp. is a wood rotting basidiomycetes and laccase is the dominant ligninolytic enzyme synthesized by this species. Laccase and other ligninolytic activities have been previously reported to be related to the stationary phase of growth in different fungi and are often triggered due to nutrient limitation [24–26]. Attempts were made to increase laccase production by the addition of the reported laccase inducer like tannic acid to enhance the expression of laccase gene at the transcription level in the growth media which showed mixed results [27]. The activity of laccase obtained from the isolated strain without inducer is comparable with that seen in *T. versicolor *without inducer, [28, 29] and *Trametes trogii *with copper as inducer [30].

Two potential inhibitors (sodium azide and EDTA) were evaluated to test the inhibition properties of laccase. Sodium azide was the most efficient inhibitor with Ki of 0.03 mM and EDTA inhibited laccase to a lesser extent with a Ki of 3.2 mM which is similar to that of laccases from *Chaetomium thermophilum *[16].

The optimal temperature range for fungal laccase activity was ranging from 30° to 60°C [31–33]. Laccase examined in this study had an optimal temperature range of 30°–65°C which is similar to that of values obtained for laccases from *Sclerotium rolfsii SRL *[34]. Generally growth of the fungi is ideal at low pH. The partially purified enzyme has an optimum pH of 4.5 for ABTS as substrate, which is comparable to the values obtained from *Tricholoma giganteum *[35] and pH 6 for syringaldazine which is comparable to *Trametes villosa *[36] and *Polyporus anceps *[37].

The SDS-PAGE results showed that *Pleurotus* spp. laccase has a molecular weight of 40 ± 1 kDa, close to that of laccases from *P. chrysosporium *having molecular weight of 46.5 kD which is in the range reported for other basidiomycetes [38]. Molecular weights of most fungal laccase proteins fall between 43,000 and 110,000 Daltons [2, 39]. A majority of laccases from basidiomycete fungi were reported to have molecular weights in the range of 55,000 to 72,000 Daltons [2, 37].

The parameters *K*_*m*_ and *V*_max_ for ABTS of partially purified laccase from *Pleurotus *sp. were 0.25 mM and 0.33 *μ*moL/min, respectively, which is very similar to that of laccase from *Melanocarpus albomyces *(0.28 mM). The *K*_*m*_ and *V*_max_ values for phenolic compound such as 2,6-DMP were 0.038 mM and 20 *μ*mol/min which are near to that of *Trametes *spp., AH28-2-A which is (0.025 mM) [38]. For nonphenolic ABTS *K*_*m*_ values around 0.03–0.05 mM have been observed for most fungal laccases [16]. On the other hand, the *K*_*m*_ values for phenolic compound 2,6-DMP were very low indicating that phenolic compounds are better substrates for *Pleurotus *sp. laccase than commonly used ABTS.

## 5. Conclusion

The fungi *Pleurotus *sp. extracellular laccase was purified from the culture filtrate of the soil isolate during two-step purification and subsequently characterized against pH 4.5 and temperature 65°C, and laccase was stable at 60°C for 8 hrs and enzyme activity was unaltered for most of the ions except Hg^+2^. The inhibitors like SDS and sodium azide completely inhibited enzyme activity. The molecular mass of the enzyme was determined to be 40 ± 1 kDa with an 72-fold and 22.4% yield. The purified enzyme had an *K*_*m*_ and *V*_max_ of 250 (mM) and 0.33 (*μ*mol/min), respectively, for ABTS as substrate where as *K*_*m*_ values for phenolic compound 2,6-DMP were 38.46 mM and *V*_max_ 20 (*μ*mol/min). The statistical optimization of a suitable medium to increase the production level of the enzyme and the factors that affect the bioelimination of a number of industrial phenolic compounds and can be detected by developing biosensor with the pure laccase are currently under progress.

## Figures and Tables

**Table 1:** Summary of purification of laccase from *Pleurotus *sp.

Purification step	Volume (mL)	Protein (mg)	Total activity (U)	Specific activity (U/mg)	Purification (fold)	Yield (%)
Culture filtrate	800	2300	29000	36	1	100
Ammonium sulfate fraction	60	1300	18000	107	2.97	62
DEAE Cellulose chromatography	15	125	14500	1300	12.14	50
Sephadex G-100 chromatography	8	12	6500	2600	72.2	22.4

**Table 2:** Effect of metal ions on purified laccase enzyme from *Pleurotus *sp.

Metal ions (2 mM)	Relative activity (%)
CuSO_4_	90
BaCl_2_	72
MgCl_2_	75
MnCl_2_	40
FeCl_2_	55
HgCl_2_	25
ZnCl_2_	41

**Table 3:** Effect of inhibitors and detergents on purified laccase enzyme from *Pleurotus *sp.

Inhibitors/detergents	Relative activity (%)
SDS (0.1%)	0
Sodium azide	10
Urea (5 M)	35
EDTA (10 mM)	65
PCMB (1 mM)	76
DTT	71
Mercaptoethanol (0.1%)	84
